# Decay of enveloped SARS-CoV-2 and non-enveloped PMMoV RNA in raw sewage from university dormitories

**DOI:** 10.3389/fmicb.2023.1144026

**Published:** 2023-04-28

**Authors:** Ye Li, K. T. Ash, Dominique C. Joyner, Daniel E. Williams, I. Alamilla, P. J. McKay, C. Iler, B. M. Green, F. Kara-Murdoch, C. M. Swift, Terry C. Hazen

**Affiliations:** ^1^Department of Civil and Environmental Engineering, University of Tennessee, Knoxville, TN, United States; ^2^Biosciences Division, Oak Ridge National Laboratory, Oak Ridge, TN, United States; ^3^Center for Environmental Biotechnology, University of Tennessee, Knoxville, TN, United States; ^4^Student Health Center, University of Tennessee, Knoxville, TN, United States; ^5^Department of Facilities Services, The University of Tennessee, Knoxville, TN, United States; ^6^Department of Earth and Planetary Sciences, University of Tennessee, Knoxville, TN, United States; ^7^Department of Microbiology, University of Tennessee, Knoxville, TN, United States; ^8^Bredesen Center, University of Tennessee, Knoxville, TN, United States; ^9^Institute for a Secure and Sustainable Environment, University of Tennessee, Knoxville, TN, United States

**Keywords:** SARS-CoV-2, PMMoV RNA, SARS-CoV-2 RNA, raw sewage, temperature, viral density

## Abstract

**Introduction:**

Although severe acute respiratory syndrome coronavirus-2 (SARS-CoV-2) RNA has been frequently detected in sewage from many university dormitories to inform public health decisions during the COVID-19 pandemic, a clear understanding of SARS-CoV-2 RNA persistence in site-specific raw sewage is still lacking. To investigate the SARS-CoV-2 RNA persistence, a field trial was conducted in the University of Tennessee dormitories raw sewage, similar to municipal wastewater.

**Methods:**

The decay of enveloped SARS-CoV-2 RNA and non-enveloped Pepper mild mottle virus (PMMoV) RNA was investigated by reverse transcription-quantitative polymerase chain reaction (RT-qPCR) in raw sewage at 4°C and 20°C.

**Results:**

Temperature, followed by the concentration level of SARS-CoV-2 RNA, was the most significant factors that influenced the first-order decay rate constants (*k*) of SARS-CoV-2 RNA. The mean *k* values of SARS-CoV-2 RNA were 0.094 day^−1^ at 4°C and 0.261 day^−1^ at 20°C. At high-, medium-, and low-concentration levels of SARS-CoV-2 RNA, the mean *k* values were 0.367, 0.169, and 0.091 day^−1^, respectively. Furthermore, there was a statistical difference between the decay of enveloped SARS-CoV-2 and non-enveloped PMMoV RNA at different temperature conditions.

**Discussion:**

The first decay rates for both temperatures were statistically comparable for SARS-CoV-2 RNA, which showed sensitivity to elevated temperatures but not for PMMoV RNA. This study provides evidence for the persistence of viral RNA in site-specific raw sewage at different temperature conditions and concentration levels.

## 1. Introduction

Wastewater-based epidemiology (WBE) of severe acute respiratory syndrome coronavirus-2 (SARS-CoV-2) is monitored from a large scale of wastewater treatment plants (Sherchan et al., 2020; Ahmed et al., 2020a, 2021), hospital sewers (Zhang et al., 2020; Gonçalves et al., 2021), rivers (Haramoto et al., 2020; Rimoldi et al., 2020), natural lakes (Gerrity et al., 2021), to a small scale of residential buildings (Wong et al., 2021), and university dormitories (Betancourt et al., 2021; Scott et al., 2021; Jain et al., 2022; Mangwana et al., 2022), the latter of which is the focus of the current study. As the wastewater from university dormitories is mainly generated from washing and bathing and sometimes contains kitchen wastewater, while the contributor to influent sewage treatment wastewater is from residential (private residences, dormitories, hotels, and residential care facilities) and commercial facilities (including hospitals), which is much more complex than the wastewater from university dormitories. Apart from the wastewater composition between university dormitories and wastewater treatment plants, the bias also appears in evaluating the decay rate of SARS-CoV-2 RNA due to the duration between excretion in feces and wastewater sampling.

Recently, most research has focused on the persistence of SARS-CoV-2 RNA in wastewater influents at different temperatures. Ahmed et al. (2020b) found that the first-order decay rate constants (*k*) of SARS-CoV-2 RNA were 0.084 day^−1^ at 4°C, 0.114 day^−1^ at 15°C, 0.183 day^−1^ at 25°C, and 0.286 day^−1^ at 37°C in wastewater. It has been observed that the decay of SARS-CoV-2 RNA happened at 4°C over 28 days but not at −20°C or −75°C (Hokajärvi et al., 2021). Furthermore, some researchers investigated the infectivity of SARS-CoV-2, the decay rate of which was much faster than its RNA decay in the wastewater influents (De Oliveira et al., 2021; Hokajärvi et al., 2021). However, these studies were conducted with spiking exogenous SARS-CoV-2, which could not completely illustrate the natural decay of SARS-CoV-2 in wastewater. Wurtzer et al. (2021) pointed out that there was not only one form of SARS-CoV-2 RNA present in wastewater, and the intact structure of the spiking SARS-CoV-2 might lead to slower decay in wastewater. Only Weidhaas et al. (2021) and Yang et al. (2022) conducted studies with endogenous SARS-CoV-2 RNA. Yang et al. (2022) found that the *k* values of endogenous SARS-CoV-2 RNA were 0.134 day^−1^ at 4°C and 0.274 day^−1^ at 26°C. Interestingly, much higher *k* values of the endogenous SARS-CoV-2 RNA were observed in the study of Weidhaas et al. (2021), in which the *k* values ranged from 2.16 to 4.32 day^−1^ at 4°C to 35°C in wastewater. However, relatively little is known about the persistence of endogenous SARS-CoV-2 RNA in raw sewage from the specific site, which could infer if the decay of SARS-CoV-2 RNA happened in-sewer travel time at some temperature conditions.

Pepper mild mottle virus (PMMoV) and SARS-CoV-2 are single-strand RNA viruses, while PMMoV is a non-enveloped virus, and SARS-CoV-2 is an enveloped virus wrapped with the phospholipid layer. It has been reported that the reduction of the enveloped virus was more efficient than non-enveloped ones due to the protection of the genome from degradation by the capsid (Ye et al., 2016). In recent years, some studies have aimed to use models to normalize the concentration of SARS-CoV-2 RNA by the concentration of PMMoV RNA to find the relationship between the concentration of SARS-CoV-2 RNA in wastewater and COVID-19 incident cases. Some parameters the models needed could be directly obtained from the SARS-CoV-2 related experiments, like flow rate and the decay rate of SARS-CoV-2 RNA; others need to infer from PMMoV RNA concentration, like fecal loads and viral shedding rates. Hence, it is also essential to study the decay of PMMoV RNA to rectify the variation of RNA recovery between samples and to detect the decay of RNA during transport and storage. Many studies focused on finding the relationship between the RNA concentrations of these two viruses, but limited information on PMMoV RNA persistence in raw wastewater compared to SARS-CoV-2 RNA. Kitajima et al. (2018) have shown that there was slight decay of PMMoV RNA in wastewater with seasonal variation. Sala-Comorera et al. (2021) found that PMMoV RNA was stable and persisted longer in aquatic environments with no significant decay. Burnet et al. (2023) highlighted no decay signal of PMMoV RNA in wastewater influent at all temperature conditions (4°C, 12°C, and 20°C) over 1 month.

Critical gaps exist in our understanding of the persistence of enveloped SARS-CoV-2 RNA and non-enveloped PMMoV RNA in site-specific raw sewage. We investigated the *k* values of these two viral RNA in raw sewage from the university dormitories using reverse transcription-quantitative polymerase chain reaction (RT-qPCR) for at least 29 days, with attention to the effects of representative temperatures in cold and temperate regions (4°C and 20°C) and different concentration levels of SARS-CoV-2 RNA (10^2^ to 10^4^ copies/L). This study could enhance further understanding of the persistence of enveloped SARS-CoV-2 and non-enveloped PMMoV RNA in the real site-specific raw sewage system. The original surveillance study at this university (Ash et al., 2023) was used to identify select samples that would provide an array of initial concentrations used for these long-term decay studies.

## 2. Materials and methods

### 2.1. Sewage sampling

During the summer of 2020, aiming to gradually end working remotely for both students and employees, the University of Tennessee administration decided to apply WBE for the early detection of COVID-19 cases in student dorms. Since each dorm has a known student number, these students could represent the health situation of the overall campus. Sewage samples were collected weekly to monitor 18 dorms, 15 Fraternities, and 14 Sororities, from 14 September 2020 to 21 September 2021, to track SARS-CoV-2 RNA in sewage during the academic semester. In order to sample from specific buildings with defined student populations, samples were collected from the downstream of dispense valves or sewer manholes just before being merged or mixed with other sewer lines. Grab samples (>50 mL) were collected from the manhole using a stainless-steel telescopic rod pole swivel dipper water swing sampler and submerged into the flowing sewage or using a sterile Nalgene bottle to collect sewage from the valve. Samples collecting started at 8:00 am. All samples were transported to the BSL-2 laboratory in a cooler containing ice within 3 h for immediate processing.

### 2.2. Sample processing

The initial concentrations of SARS-CoV-2 and PMMoV RNA were determined immediately by 50 ml of well-mixed raw sewage samples at time zero (*t* = 0) within 5 h of sample collection. Remain samples were incubated in the dark at 4°C and 20°C to evaluate the temperature effect on the decay of SARS-CoV-2 and PMMoV RNA in sewage. On specific days, sewage samples were retrieved from each temperature condition.

Sewage samples were pasteurized for 2 h at 60°C, followed by centrifuging at 5,000 × g for 10 min, and then filtration through a 0.45 and 0.22 μm nitrocellulose filter to remove large suspended particulate matter. The filtered samples were concentrated using ultrafiltration with an Amicon Ultra-15 filtration device (EMD Millipore, Burlington, MA). Centrifuge the Amicon Ultra at 4,000 × g for 30 min (Swing-arm rotor) or 5,000 × g for 20 min (Fixed-angle rotor) at room temperature. The concentrate was transferred to (~250 μL) 2 mL DNA LoBind tubes, and RNA exactions were carried out using a Qiagen viral RNA Mini Kit (Qiagen, Valencia, CA, United States; Ash et al., 2021). Briefly, a volume of 60 μL of RNA was extracted using the Qiagen viral RNA Mini Kit following the instructions of the manufacturer from a homogenized sample. DNase/RNase-free water was used as extraction negative control. All RNA samples were stored at −80°C and subjected to RT-qPCR analysis within 1 day of RNA extraction.

### 2.3. RT-qPCR

RT-qPCR was used to quantify the concentrations of SARS-CoV-2 and PMMoV RNA in each sample. CDC primer/probe assays SARS-CoV-2 N1was used, which was quantified using the TaqPath 1-Step RT-qPCR Master Mix, CG (Thermo Fisher Scientific) on an Applied Biosystems QuantStudios 7 Pro Real-Time PCR System instrument (Ash et al., 2023). Each 20 μL reaction contained 5 μL of 4X Master Mix (Thermo Fisher Scientific), 0.25 μL of 10 μmol/L probe, 1 μL each of 10 μmol/L forward and reverse primers, 7.75 μL of nuclease-free water, and 5 μL of nucleic acid extract. Reagents were pipetted into 96-well plates and vortexed for 10 s. Thermocycling conditions were as follows: uracil-DNA glycosylase incubation for 2 min at 25°C, reverse transcription for 15 min at 50°C, activation of the Taq enzyme for 2 min at 95°C, and two-step cycling for 3 s at 95°C and 30 s at 55°C for 45 cycles. A positive test result was defined as an exponential fluorescent curve that crossed the threshold within 40 cycles (cycle threshold [C_t_] <40).

PMMoV was also quantified by RT-qPCR using the TaqPath 1-Step RT-qPCR Master Mix, CG (Thermo Fisher Scientific) on a QuantStudios 7 Pro instrument. Each 20 μL reaction contained 5 μL of 4X Master Mix (Thermo Fisher Scientific), 0.5 μl of 10 μmol/L probe, 1.8 μL each of 10 μmol/L forward and reverse primers, 8.9 μL of nuclease-free water, and 2 μL of nucleic acid extract. Reagents were pipetted into 96-well plates and vortexed for 10 s. Thermocycling conditions were as follows: uracil-DNA glycosylase incubation for 2 min at 25°C, reverse transcription for 15 min at 50°C, activation of the Taq enzyme for 10 min at 95°C, and two-step cycling for 30 s at 95°C and 1 min at 60°C for 40 cycles.

A series of three positive and negative controls (Mastermix + DNase/RNase-free water) were included in each RT-qPCR run. All RT-qPCR reactions were performed in triplicate. The results were applied only if the positive control was positive and the negative control was negative. The sample was defined as positive only if all replicates were positive, each of which should be within the linear range of the standard curve. The efficiency of the N1 standard curve was 94.669% (R^2^ = 1). The final quantification of SARS-CoV-2 RNA was the mean of three replicates of virus copies. RT-qPCR outputs were converted to copies per liter. The detection limit of SARS-CoV-2 and PMMoV was 20 and 10 copies/L in this study.

### 2.4. Data analysis

The *k* and T_90_ were calculated for each temperature in Sigma Plot 14_5 by Eqs. (1, 2), respectively.


(1) $$C_{t}=C_{0}e^{-kt}$$

(2) $$T_{90}=-\frac{\ln\left(0.1\right)}{k}$$

where *k* is the first-order decay rate. C_t_ and C_0_ are the concentration of the viral RNA at time t and time zero, respectively. T_90_ is the days needed to achieve a 90% reduction in initial concentration. The fit of the model is evaluated by r^2^.

In this study, the significant factors on *k* values were determined by a multiple linear regression model [Eq. (3)]. It could explain the impact of influential variables (temperature, concentration levels of SARS-CoV-2, decay rate constant of PMMoV RNA, and sampling sites) as independent data on the first decay rate constants of *k*, which also included the interaction effects between different influential variables.


(3) $$\log_{10}\;k=a+\sum_{i}^{n}b_{i}x_{i}+\varepsilon =a+b_{1}x_{1}+b_{2}x_{2}+\ldots +\varepsilon$$

where x_i_ is the influential variable, a is the intercept, *b_i_* is the regression coefficient, and *ε* is the regression residual.

All linear fitting and statistical analyses were performed using SPSS (version 26). One-way ANOVA with the Kruskal–Wallis H test was used to evaluate the effect of different SARS-CoV-2 concentration levels and sampling sites, as well as the Mann–Whitney U test to compare differences between different temperatures. The paired *t*-test was used to compare the decay of SARS-CoV-2 and PMMoV RNA (α = 0.05 for both tests). All statistical differences were determined by *p* < 0.05.

## 3. Results

### 3.1. The decay rate and multiple linear regression models

The first-order decay rate model fits most decay curves of SARS-CoV-2 RNA (r^2^ = 0.512 to 0.996; Table 1), a bad fit for most decay curves of PMMoV RNA (r^2^ = 0 to 0.996; Table 1). The decay curves with smaller r^2^ had smaller *k* values, indicating limited decay with time. The *k* and T_90_ for SARS-CoV-2 and PMMoV RNA are shown in Table 1.

**Table 1:** The *k* and T_90_ values of SARS-CoV-2 and PMMoV RNA in raw sewage at 4°C and 20°C.

Sample	T (°C)	SARS-CoV-2 RNA					PMMoV RNA			
		C_0_ (Copies/L)	Con. Level	*k* (day^−1^)	r^2^	T_90_ (days)	C_0_ (Copies/L)	*k* (day^−1^)	r^2^	T_90_ (days)
F4	4	4.14E + 04	High	2.25E − 02	0.717	1.02E + 02	1.87E + 05	1.57E − 13	0.000	1.84E + 01
	20			1.25E − 01	0.701	1.84E + 01		7.00E − 04	0.001	3.29E + 03
D13-6	4	2.10E + 04	High	4.97E-01	0.804	4.63E + 00	7.52E + 04	1.06E − 02	0.115	2.17E + 02
	20			3.90E − 01	0.987	5.91E + 00		3.25E − 02	0.890	7.08E + 01
D13-1	4	1.14E + 04	High	4.20E − 01	0.904	5.48E + 00	1.81E + 05	4.33E − 02	0.283	5.32E + 01
	20			7.47E − 01	0.996	3.08E + 00		5.26E − 13	0.000	4.38E + 12
D13-24-1	4	6.42E + 03	Median	1.16E − 01	0.910	1.98E + 01	2.57E + 05	5.61E − 01	0.677	4.11E + 00
	20			7.63E − 01	0.980	3.02E + 00		5.53E − 01	0.636	4.17E + 00
D3	4	3.69E + 03	Median	8.50E − 03	0.647	2.71E + 02	1.40E + 06	9.20E − 03	0.034	2.50E + 02
	20			3.31E − 01	0.983	6.97E + 00		6.58E − 01	0.996	3.50E + 00
S14	4	1.84E + 03	Median	7.36E − 02	0.987	3.13E + 01	7.15E + 04	2.99E − 02	0.584	7.70E + 01
	20			2.65E − 01	0.990	8.70E + 00		1.79E − 02	0.324	1.29E + 02
D13-2-1	4	1.82E + 03	Median	2.83E − 02	0.591	8.14E + 01	1.73E + 05	2.15E − 12	0.000	1.07E + 12
	20			3.16E − 01	0.954	7.28E + 00		1.22E − 02	0.207	1.89E + 02
D13-24-2	4	1.57E + 03	Median	1.65E − 02	0.700	1.40E + 02	1.45E + 05	1.51E − 13	0.000	1.52E + 13
	20			3.72E − 02	0.982	6.19E + 01		3.56E − 13	0.000	6.47E + 12
S12-1	4	1.08E + 03	Median	1.36E − 02	0.824	1.69E + 02	3.38E + 05	8.30E − 03	0.015	2.77E + 02
	20			6.07E − 02	0.833	3.79E + 01		3.88E − 11	0.000	5.93E + 10
F7	4	9.24E + 02	Low	2.53E − 02	0.880	9.10E + 01	9.96E + 04	7.61E − 13	0.000	3.02E + 12
	20			3.13E − 01	0.940	7.37E + 00		2.76E − 11	0.000	8.34E + 10
S12-2	4	7.84E + 02	Low	8.60E − 03	0.895	2.68E + 02	5.66E + 05	2.69E − 13	0.000	8.57E + 12
	20			1.29E − 02	0.812	1.78E + 02		2.70E − 03	0.085	8.53E + 02
D13-2-2	4	3.10E + 02	Low	1.02E − 01	0.885	2.26E + 01	4.04E + 05	4.02E − 02	0.127	5.73E + 01
	20			1.92E − 01	0.823	1.20E + 01		4.66E − 02	0.496	4.94E + 01
F2	4	2.59E + 02	Low	2.95E − 02	0.512	7.81E + 01	6.65E + 04	4.42E − 02	0.623	5.21E + 01
	20			1.14E − 01	0.801	2.02E + 01		6.25E − 12	0.000	3.68E + 11
D14-1	4	1.92E + 02	Low	1.51E − 02	0.615	1.52E + 02	2.38E + 05	1.70E − 03	0.002	1.35E + 03
	20			1.24E − 01	0.893	1.86E + 01		1.50E − 03	0.003	1.54E + 03
D14-2	4	1.89E + 02	Low	3.52E − 02	0.739	6.54E + 01	1.64E + 05	4.25E − 12	0.000	5.41E + 11
	20			1.17E − 01	0.646	1.98E + 01		6.30E − 03	0.050	3.65E + 02
NC	4	1.62E + 03	-	2.11E − 01	0.650	1.09E + 01	2.20E + 06	9.00E − 03	0.384	2.56E + 02
CL	4	1.34E + 02	-	5.00E − 03	0.688	4.61E + 02	1.33E + 06	4.28E − 13	0.000	5.38E + 12

The multiple regression model revealed that the influential variables, e.g., temperature, PMMoV RNA, concentration levels of SARS-CoV-2 RNA, and the interaction between temperature and concentration levels of SARS-CoV-2 were significant for the first decay rate constants [*F* (4, 29) = 9.084, *p*-value < 0.0001]. The sampling site was removed from the model due to no significance. The modified model indicated that temperature, PMMoV RNA, and the concentration levels of SARS-CoV-2 RNA remained significant (t = 3.850, 2.185, −2.817, and the corresponding *p*-value was 0.001, 0.009, and 0.038, respectively) but not the interaction term, suggesting that the effect of temperature is rarely dependent on the concentration levels of SARS-CoV-2. Therefore, the regression model only included temperature, PMMoV RNA, and the concentration levels of SARS-CoV-2 as influential variables. The intercept (*a*) and coefficients (*b*_temperature_, *b*_log10 kPMMoV_, and *b*_concentration level of SARS-CoV-2_) of the final regression model were − 0.714, 0.039, 0.035, and − 0.304, respectively. The *F*-ratio in the ANOVA table showed that the independent variables significantly predicted the dependent variable, *F* (3, 27) = 10.767, *p*-value < 0.0001. The positive regression coefficient indicated that the first decay rate constants increased with temperature and were higher at the high decay rate of PMMoV. The negative regression coefficient indicated that *k* was higher at the low-concentration level of SARS-CoV-2 than at the high level.

### 3.2. The *k* and T_90_ values of SARS-CoV-2 RNA at different concentration levels of SARS-CoV-2 and various temperatures

**Figure 1:**
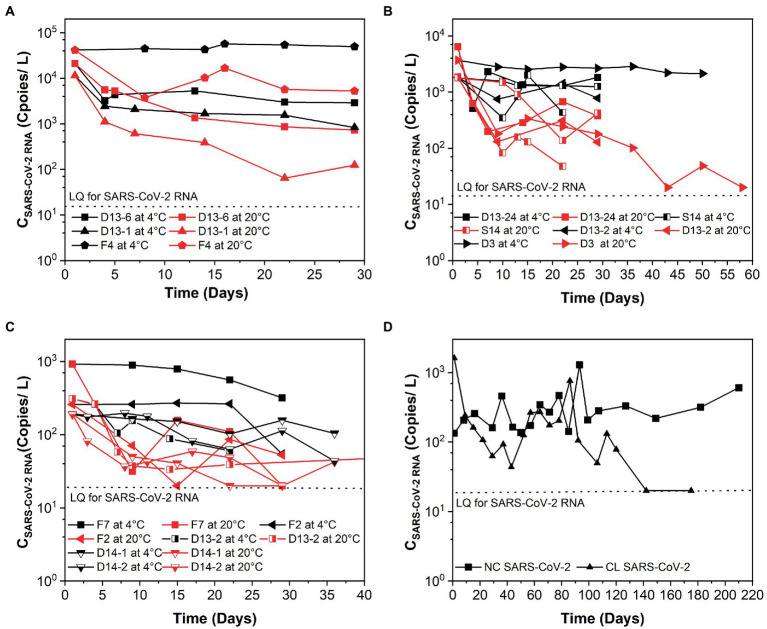
Persistence of SARS-CoV-2 over time (days) in raw sewage from university dormitories. **(A)** High-concentration level of SARS-CoV-2 (C_SARS-CoV-2 RNA_ > 10^4^ copies/L), **(B)** Medium-concentration level of SARS-CoV-2 (10^4^ copies/L > C_SARS-CoV-2 RNA_ > 10^3^ copies/L), **(C)** Low-concentration level of SARS-CoV-2 (10^3^ copies/L > C_SARS-CoV-2 RNA_ > 10^2^ copies/L), **(D)** Long time decay of SARS-CoV-2 at 4°C. LQ means the limit of quantification in RT-qPCR. F4, D13-6, D13-1, D13-24-1, D3, S14, D13-2-1, D13-24-2, S12-1, F7, S12-2, D13-2-2, F2, D14-1, D14-2, NC, and CL correspond to different campus samples.

The *k* and T_90_ values of SARS-CoV-2 RNA are summarized in Table 1, and the decay curves of SARS-CoV-2 RNA are shown in Figure 1. The average initial concentrations (mean ± standard deviation) of SARS-CoV-2 RNA from high to low concentration levels ranged from 4.39 ± 4.19 to 2.65 ± 2.51 log_10_ copies/L. The mean *k* values were 0.367, 0.169, and 0.091 day^−1^ with the corresponding T_90_ values of 6.27, 13.62, and 25.30 days at high-, medium-, and low-concentration levels of SARS-CoV-2 RNA, respectively. The *k* values from high to low concentration levels of SARS-CoV-2 in sewage were significantly decreased (Kruskal–Wallis H = 7.48, *p*-value = 0.024). The temperature remained stable at 4.0°C ± 1°C and 20°C ± 2°C throughout the experiment. The mean *k* values were 0.094 day^−1^ at 4°C and 0.261 day^−1^ at 20°C. The decay characteristic of SARS-CoV-2 RNA at high temperature (20°C) was significantly faster compared to the low temperature (4°C; Mann–Whitney *U* = 59.00, *p*-value = 0.003). In addition, the mean T_90_ values of the SARS-CoV-2 RNA in raw sewage were 24.47 and 8.84 days, corresponding to 4°C and 20°C. When comparing decay rate constants among different concentration levels of SARS-CoV-2 at the same temperature, there were no significant differences for both viral RNA at 4 and 20°C (Table 1). The mean *k* values for SARS-Co-2 RNA at 4 and 20°C were 0.313 and 0.421 day^−1^ at the high-concentration level of SARS-CoV-2, 0.043 and 0.295 day^−1^ at the median concentration level, and 0.036 and 0.145 day^−1^ at the low-concentration level. The T_90_ values were 37.37, 118.75, and 120.87 days at 4°C and 9.13, 20.96, and 42.66 days at 20°C corresponding to the concentration levels of SARS-CoV-2 RNA at high, medium, and low, respectively. The decay of high-concentration levels of SARS-CoV-2 RNA was much faster than that with low-concentration levels of SARS-CoV-2 RNA at both low and high temperatures in raw sewage.

### 3.3. The *k* and T_90_ values of PMMoV RNA at different concentration levels of SARS-CoV-2 and various temperatures

**Figure 2:**
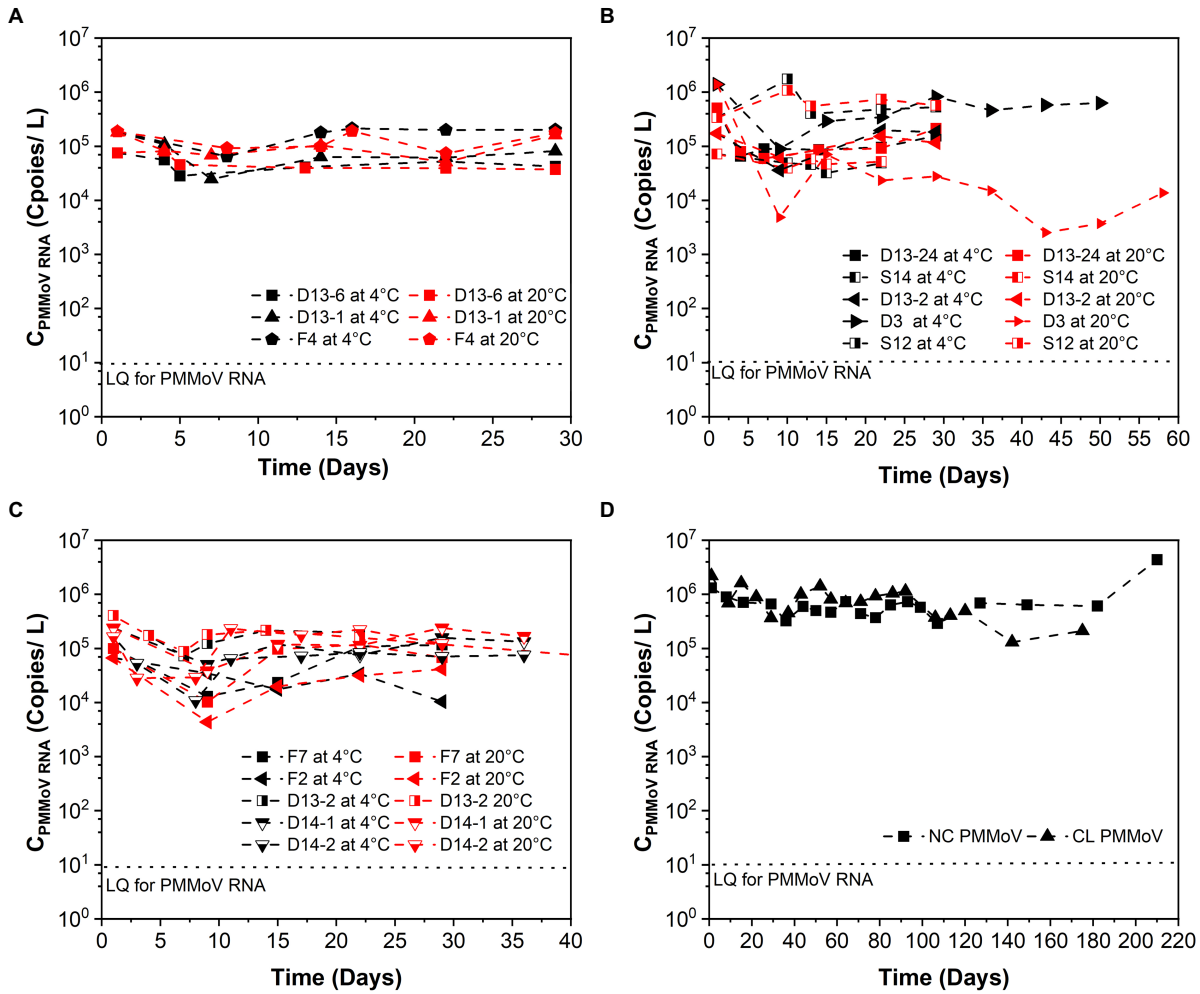
Persistence of PMMoV RNA over time (days) in raw sewage from university dormitories. **(A)** High-concentration level of SARS-CoV-2 (C_SARS-CoV-2 RNA_ > 10^4^ copies/L), **(B)** Medium-concentration level of SARS-CoV-2 (10^4^ copies/L > C_SARS-CoV-2 RNA_ > 10^3^ copies/L), **(C)** Low-concentration level of SARS-CoV-2 (10^3^ copies/L > C_SARS-CoV-2 RNA_ > 10^2^ copies/L), **(D)** Long time decay of SARS-CoV-2 at 4°C. LQ means the limit of quantification in RT-qPCR. F4, D13-6, D13-1, D13-24-1, D3, S14, D13-2-1, D13-24-2, S12-1, F7, S12-2, D13-2-2, F2, D14-1, D14-2, NC, and CL correspond to different campus samples.

Table 1 summarizes the *k* and T_90_ values of PMMoV RNA, and Figure 2 shows the persistence of PMMoV RNA in raw sewage. The mean initial concentrations (mean ± standard deviation) of PMMoV RNA ranged from 5.17 ± 4.80 to 5.60 ± 5.70 log_10_ copies/L at different concentration levels of SARS-CoV-2 RNA from high to low. The decay of PMMoV RNA was limited at all concentration levels of SARS-CoV-2 over at least 29 days. The mean *k* values ranged from 0.012 to 0.154 day^−1^ with T_90_ values from 14.95 to 191.88 days at different concentration levels of SARS-CoV-2 RNA, with no statistical difference. The mean *k* values were 0.05 day^−1^ at 4°C and 0.09 day^−1^ at 20°C, with the corresponding T_90_ values of 46.15 and 25.94 days. The decay characteristic of PMMoV RNA at different temperatures showed no statistical difference.

In contrast to SARS-CoV-2 RNA, PMMoV RNA persisted for longer periods at both temperatures evaluated. The mean *k* value for PMMoV RNA at 4 and 20°C was 0.018 and 0.011 day^−1^ at the high-concentration level of SARS-CoV-2, 0.101 and 0.207 day^−1^ at the median concentration level, and 0.014 and 0.010 day^−1^ at the low-concentration level, respectively. T_90_ values of PMMoV RNA were 127.92, 22.80, and 164.47 days at 4°C and 209.33, 11.12, and 230.26 days at 20°C from the high to the low concentration level of SARS-CoV-2, respectively.

### 3.4. The *k* values of SARS-CoV-2 vs. PMMoV RNA

Viral RNA from SARS-CoV-2 and PMMoV remained detectable for at least 29 days. SARS-CoV-2 RNA was relatively stable with faster decay rates at 20°C than at 4°C in our study. In contrast to SARS-CoV-2, PMMoV persisted for longer periods at both temperature conditions. The decay rates for SARS-CoV-2 and PMMoV were significantly different at both temperatures evaluated (paired *t*-test, *t* = 3.733, *p*-value < 0.002, at 4°C and paired *t*-test, t = 3.441, *p*-value < 0.004, at 20°C). In addition, we noted that regardless of other factors, there was a significant difference between the decay characteristics of SARS-CoV-2 and PMMoV RNA (paired *t*-test, t = 5.153, *p*-value < 0.001).

## 4. Discussion

Several studies have reported higher persistence of SARS-CoV-2 RNA in wastewater at low temperatures compared to high temperatures, which was consistent with our research. It was observed that SARS-CoV-2 RNA could be detectable over 100 days at 4°C (Figure 1D). As Aboubakr et al. (2021) summarized that the structure of enveloped viruses like coronaviruses was fragile, and the way they infected their host cells made them susceptible to heat. As the morphology and chemical structure of the SARS-CoV-2 virus is similar to other coronaviruses, the possible explanation for this trend was reported as due to the denaturation of proteins and increased activity of extracellular enzymes under higher temperature, which facilitated the decay of SARS-CoV-2 (Aquino de Carvalho et al., 2017). Ahmed et al. (2020b) reported that the *k* values of spiked with gamma-irradiated SARS-CoV-2 were 0.084 day^−1^ at 4°C, 0.114 day^−1^ at 15°C, 0.183 day^−1^ at 25°C, and 0.286 day^−1^ at 37°C in wastewater. The decay rate constants of SARS-CoV-2 RNA in the study of Ahmed et al. (2020b) were slightly higher than our results. The reason for this could be due to the different sample preparation. Wurtzer et al. (2021) pointed out that there was not only one form of SARS-CoV-2 RNA present in sewage, including integrated viruses, genomic RNA protected within an infectious or non-infectious structure, and free total or partial genomic RNA. Yang et al. (2022) inferred that the integrity of the viral structure might be better than in raw wastewater, so the incomplete viral structure made viral RNA easier to degrade. Future study is necessary to investigate the influential elements that differentiate the decay between endogenous and exogenous SARS-CoV-2 RNA in sewage. In addition, Hokajärvi et al. (2021) conducted the study with exogenous SARS-CoV-2 RNA and showed that it decayed over 28 days at 4°C but not at −20°C or −75°C. Recently, Yang et al. (2022) observed that the *k* values of endogenous SARS-CoV-2 RNA were 0.134 and 0.274 day^−1^ at 4°C and 26°C, respectively. These values were slightly higher than our studies at similar temperature conditions. Interestingly, Weidhaas et al. (2021) obtained much higher *k* values of the endogenous SARS-CoV-2 RNA than our study, in which the *k* values ranged from 2.16 to 4.32 day^−1^ at 4 to 35°C in wastewater. This may be due to the experimental durations. From Figures 1A–C, we could also observe that at the very beginning, SARS-CoV-2 RNA decayed much faster than in the following days. In addition, Roldan-Hernandez et al. (2022) reported that the short length of the study could observe limited decay, which affected the *k*. The duration of our study was at least 29 days, but just 4 and 1 days for the studies of Hokajärvi et al. (2021) and Yang et al. (2022), respectively.

We had similar results with Bivins et al. (2020) regarding the decay rate of different SARS-CoV-2 concentrations, who revealed that the k of the high titer SARS-CoV-2 concentration was 0.67, which is around 7 times higher than the low titer decay rate in 20°C raw sewage samples over the experiment duration of 7 days, the reason for which is unknown.

The decay characteristic of PMMoV RNA at different temperatures showed no statistical difference. This was in agreement with the study of Burnet et al. (2023), who found no significant decay signal of PMMoV RNA in wastewater influent at all temperature conditions (4°C, 12°C, and 20°C) over 1 month. Kitajima et al. (2018) summarized that there was a slight seasonal variation of PMMoV RNA in wastewater. In addition, Sala-Comorera et al. (2021) found that PMMoV RNA was stable and persisted longer in aquatic environments, and no significant decline was observed. From these studies, PMMoV can be considered a conservative marker with respect to the virus reduction (Kitajima et al., 2018; Sala-Comorera et al., 2021). As PMMoV is a non-enveloped virus, it has been reported that the long persistence of the non-enveloped virus was due to lack of a structure named lipid bilayer outside the viral protein capsid that is sensitive to the detergents and organic solvents in the wastewater (Vidaver et al., 1973).

It has been reported that enveloped viruses were commonly thought to be less persistent than non-enveloped viruses due to the structure of a lipid layer, which is unstable at high temperatures and increases the decay of the virus RNA. Furthermore, the wastewater detergent and other chemical agents could be penetrated the lipid layer, which led to the degrading of the viral envelope (Wurtzer et al., 2021).

## 5. Conclusion

This study conducted a field trial on the persistence of enveloped SARS-CoV-2 and non-enveloped PMMoV RNA in raw sewage from university dormitories. In our study, SARS-CoV-2 RNA decayed significantly faster at 20°C than at 4°C. The decay rate constants of the high-concentration levels of SARS-CoV-2 RNA were significantly higher than the lower levels at both temperature conditions and could be detected at least 29 days at all concentration levels. Due to the prolonged persistence of SARS-CoV-2 RNA at 4°C, it suggested that sewage samples could be stored at 4°C without significant degradation for a considerable length of time. This is particularly important for laboratories where resources and throughput are limited, and sample storage is unavoidable. We also need to pay attention to the decay of SARS-CoV-2 RNA at higher concentration levels and higher temperatures for long in-sewer traveling time. In contrast to enveloped SARS-CoV-2 RNA, non-enveloped PMMoV RNA could be highly persistent in raw sewage at both temperature conditions. Future studies will need to identify why the decay of SARS-CoV-2 RNA at different concentration levels is significant.

## Data availability statement

The original contributions presented in the study are included in the article/supplementary material, further inquiries can be directed to the corresponding author.

## Author contributions

YL and TH: writing. DW, IA, PM, DJ, and CI: collection. YL, KA, DW, DJ, IA, PM, CI, and BG: lab analysis. YL, KA, and TH: data Analysis. TH and DJ: project management. All authors contributed to the article and approved the submitted version.

## Conflict of interest

The authors declare that the research was conducted in the absence of any commercial or financial relationships that could be construed as a potential conflict of interest.

## Publisher’s note

All claims expressed in this article are solely those of the authors and do not necessarily represent those of their affiliated organizations, or those of the publisher, the editors and the reviewers. Any product that may be evaluated in this article, or claim that may be made by its manufacturer, is not guaranteed or endorsed by the publisher.
